# The neurodevelopmental basis of humor appreciation: A fNIRS study of young children

**DOI:** 10.1371/journal.pone.0259422

**Published:** 2021-12-08

**Authors:** Naama Mayseless, Allan L. Reiss

**Affiliations:** 1 Center for Interdisciplinary Brain Sciences Research, Department of Psychiatry and Behavioral Sciences, Stanford University School of Medicine, Stanford, CA, United States of America; 2 Departments of Radiology and Pediatrics, Stanford University School of Medicine, Stanford, CA, United States of America; Tokai University, JAPAN

## Abstract

Humor is crucial for social development. Despite this, very few studies have examined the neurodevelopment of humor in very young children, and none to date have used functional near-infrared spectroscopy (fNIRS) to study this important cognitive construct. The main aim of the current study was to characterize the neural basis of humor processing in young children between the ages of 6–8 years. Thirty-five healthy children (6–8 years old) watched funny and neutral video clips while undergoing fNIRS imaging. We observed activation increases in left temporo-occipito-parietal junction (TOPJ), inferior-parietal lobe (IPL), dorsolateral-prefrontal cortex (DLPFC) and right inferior frontal gyrus (IFG) and superior parietal lobe (SPL) regions. Activation in left TOPJ was positively correlated with age. In addition, we found that coherence increased in humor viewing compared to neutral content, mainly between remote regions. This effect was different for boys and girls, as boys showed a more pronounced increase in coherence for funny compared to neutral videos, more so in frontoparietal networks. These results expand our understanding of the neurodevelopment of humor by highlighting the effect of age on the neural basis of humor appreciation as well as emphasizing different developmental trajectories of boys and girls.

## Introduction

Humor is a universal human feature which refers to activities that are considered funny and make people laugh as well as to the processes that go into creating and perceiving these activities and the amusing feeling that arises [1]. There are several cognitive theories of humor. One of the most prominent theories of humor is the incongruity detection and resolution theory which suggests that humor requires first the introduction of incongruity, which produces an unexpected violation of expectations, and results in cognitive arousal; and incongruity resolution associated with amusement [2–4]. This is best seen in jokes that include a setup and a punch line. Wyer and Collins [5] expanded on this concept to suggest the comprehension elaboration theory which posits that humor processing relies on two phases: comprehension and elaboration [2]. According to this theory, comprehension refers to the stage of incongruity detection and resolution, whereas elaboration refers to the subsequent enjoyment that follows humor comprehension. Recently a three-stage model was suggested consisting of incongruity detection, resolution and elaboration [2, 6, 7]. These theories imply a time component in humor processing, where comprehension and appreciation of humor rely on time varying processes. Expanding on the incongruity component of humor theories, Veatch [8] formulated that humor contains two incongruous elements; one is socially normal while the other is a violation of the “subjective moral order.” Veatch defines this moral order as a cognitive and emotional system of ideas about the social and natural world order (p. 168, [8]. This view stresses the need for a logical development of expectations about a situation that must be violated for humor to be perceived. As such, when looking at the development of humor across the ages, for humor to be present, a moral development needs to occur in addition to the need for cognitive development.

Humor and a sense of humor is crucial for social development of children. Humorous encounters encourage playfulness [9], are important for development of joint attention [10] and understanding of other’s emotional attitudes, expectations and intentions [11, 12]. Humor, a cognitive affective style, comprises diverse capabilities including the ability to comprehend, appreciate, enjoy, create, and relay positive incongruous communication [13]. It plays a critical role in building and maintaining relationships, emotional health, and cognitive function [1]. For children, attaining a sense of humor is part of a normal developmental sequence that includes maturation of physical, cognitive, linguistic, and social skills [14, 15]. Humor begins as events can be stored and recalled as simple images and continues to develop as a child’s thinking becomes more conceptual and language is more evolved [16]. This developmental framework points to a maturation in types of humor that children find enjoyable, moving from slapstick humor to enjoyment of riddles and jokes based on double meanings [17]. As such, atypical development has been linked to altered sense of humor. For example, learning disabilities were associated with a lag in the ability to comprehend and appreciate humor [18]. Further, children with autism often exhibit humor appreciation and generation that is very different than typically developing children [19]. In addition, children with autism respond differently when asked to interpret incongruity, a necessary step for later appreciation of complex humor [20]. In addition to autism, schizophrenia has also been associated with altered responses to humorous content [21, 22]. Given humor’s role in development, it is especially important to understand its underlying neural basis and developmental profile. Such an understanding could potentially be used to aid in the early identification of those at risk for some neuropsychiatric conditions such as autism, schizophrenia and personality disorders, reinforcing the need for a better understanding of this important cognitive ability in childhood.

Previous imaging studies performed in adults have implicated brain regions related to the detection and resolution of incongruity in humor including the left inferior frontal gyrus, inferior temporal gyrus, the left fusiform gyrus, and the temporal-occipital-parietal junction (TOPJ), which includes Brodmann areas 37, 39, and 40 [23–25]. To date, the only studies examining the neural pathways of humor processing in children were done by our group using functional magnetic resonance imaging (fMRI) in participants 6 to 13 years of age. The stimuli used in these studies were chosen to account for the developmental stage of the participants, and included short video clips of funny, positive or neutral videos. These studies identified several brain regions involved in humor appreciation in young children including bilateral temporo-occipito-parietal areas (TOPJ), regions of the midbrain and prefrontal regions [26–28]. In our initial study [26] we examined children between the ages of 7.9 to 11.7 (15 subjects, 9 female). The main finding was the observation of neural responses to funny vs neutral clips in the left and right TOPJ. However, additional clusters were observed in the left and right occipital lobes (BA 17 and 18) as well as the right inferior parietal lobule (BA 40). We also found that age was related to activation patterns in a right-lateralized network. These results, which are similar to activation patterns seen in adults, suggest that a humor-processing network is already present in early childhood. Some differences do emerge though between adults and children. While adults typically exhibit activation lateralized to left TOPJ [29], children exhibit bilateral activations in this region suggesting that lateralization occurs during adulthood as a component of typical maturation [26].

Vrticka, Neely [28] extended these results by examining sex differences in activation patterns among 22 typically developing children (ages 6.7–13 years, 13 female). We reported that females displayed a stronger activation contrasts for funny versus positive clips in right lateralized regions (including supramarginal gyrus (SMG) and superior temporal sulcus (STS) and left TPJ. Male participants showed overall higher activity for positive versus funny clips in bilateral inferior parietal lobe (IPL, BA 40), right fusiform face area (BA 37), and right BA 46.

In a follow-up investigation [27], we showed that neural activation patterns related to humor processing in children is moderated by temperament traits such as emotionality, shyness and sociability. Overall, these studies provide initial evidence that primary regions observed to underlie humor processing in adults (e.g., bilateral TOPJ) are already established in children, however, involvement of other regions observed in adult studies seem to be related to age (IFG). While these studies shed light on humor across school-age child development, further investigations are warranted, particularly in the youngest age groups able to participate in a functional imaging study. The similarities found in these studies between activation patterns of childhood and those found in adult studies, coincide with theories of humor development that suggest emergence of adult-like humor perception at or after the age of 6 [30, 31]. According to Shultz (1976), the real appreciation of humor demands that children are able not only to represent incongruities, but also to resolve them, and this ability does not emerge before 6 years of age. It is therefore possible that results of previous imaging studies among 6–13 year old children [26] are influenced by the older aged children in the study. Accordingly, here we chose to focus on a more limited age group, 6–8 year olds, as the hypothesized transition from incongruity to incongruity plus resolution occurs around this maturational period (Shultz 1974). Studying early childhood is critical, because it is during this time period that the ability to detect and resolve incongruencies develops, accompanied by the emerging ability to regulate and manage one’s emotions [14].

In the current study, we significantly extend research knowledge of humor processing in children by utilizing functional near infrared spectroscopy (fNIRS), a methodology that allows for more portable, mobile and flexible neuroimaging than what is possible using functional magnetic resonance imaging (fMRI). Because fNIRS is noninvasive and does not require as much motion suppression as fMRI, it is especially well-suited for brain imaging studies of infants and children in naturalistic settings. Our goal was to both replicate and developmentally extend previous studies of children’s humor appreciation using fNIRS. In order to achieve this goal, we focused on younger children in a more homogeneous age group, corresponding to the beginning of public-school attendance (ages 6–8 years old). Furthermore, we were interested in extending these results using connectivity patterns to discern possible network connections within brains of children viewing humorous stimuli.

Based on previous studies we formulated the following hypotheses: 1. We anticipated activation to be consistent with previous fMRI studies showing increased activation in response to humor viewing in left TOPJ as well as IPL and IFG regions. 2. We also hypothesized that there would be a correlation between activation patterns and age in parietal areas. Specifically, we anticipated a positive correlation between activation patterns and age. 3. In addition, we anticipated an effect of sex on humor processing. We hypothesized that girls and boys would exhibit different activation patterns in accordance with sex/gender developmental theories.

## Methods

### Participants

Based on a previous study using the same task [26] and expected attrition of participants, including bad data and excessive movements, a total of 38 (female = 18) typically developing children between the ages of 6.1 and 8.7 years were recruited for participation. All participants were right-handed, had normal or corrected to normal vision and hearing, had no clinical psychiatric symptoms or problems as indicated by parental responses to medical history-related questions during a phone screening, and were of average to gifted intelligence as assessed by the Wechsler Abbreviated Scale of Intelligence, Second Edition (WASI-II). (WASI-II; [32]). Participants and parents provided assent and consent, respectively, before participation. The protocol was approved by the Stanford University Institutional Review Board, and all clinical investigation was conducted in accordance with the principles expressed in the Declaration of Helsinki. Two Participants were excluded due to noisy data, and one was excluded due to problems with event logging, resulting in a final sample of 35 participants. Participants were enrolled in kindergarten (N = 14), first grade (N = 7) and second grade (N = 14).

### Experimental task

We used a modified version of a humor appreciation and comprehension task described in Neely et al. [26] and Vrticka et al. [27]. During the task, children were asked to view short clips depicting funny and neutral scenes and events. In accordance with Vrticka et al. [28], the stimuli chosen were pilot-tested with a matched sample of children who did not participate in scanning. Funny video clips were defined as having mean ratings ≥5 on the funny scale (the scale was set as 1 = least funny to 8 = most funny); Neutral video clips were defined as having mean ratings ≤4 on both the funny and enjoyable scales (scale from 1 = least funny/enjoyable to 8 = most funny/enjoyable see also Neely et al., 2012). All videos were presented without sound and most would be classified as slapstick humor. Selected funny video clips included scenes of people stumbling while skiing or running, a kid “swimming” on the floor, cars accidentally running in to things, animals performing amusing tricks, a kid being catapulted into the air from an inflatable couch, and many others. Neutral video clips included kids riding bicycles, kids singing in school plays, nature documentary style videos of animals, and related scenes. Examples of clip can be found here: https://osf.io/436ex/.

**Fig 1:**
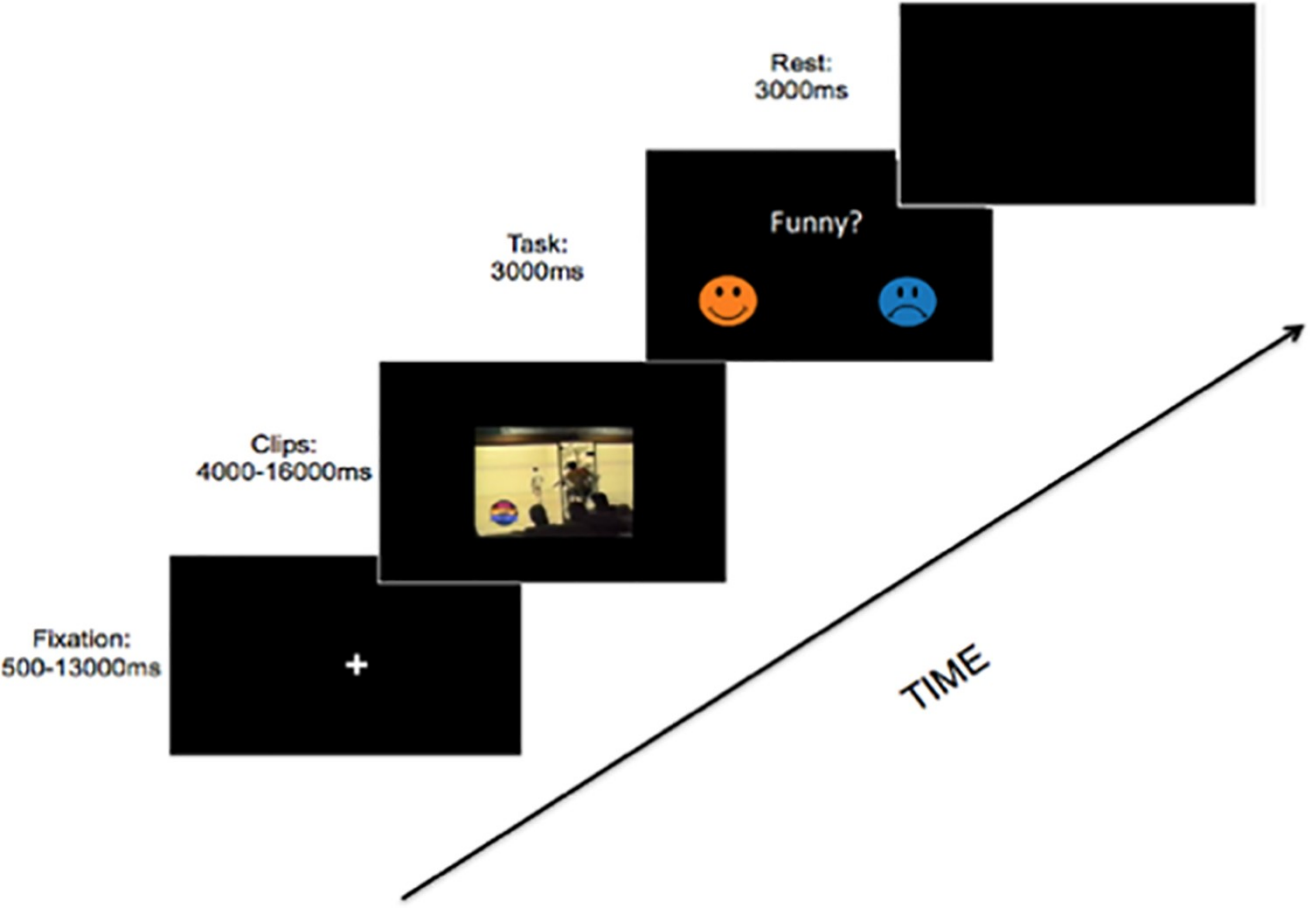
Experimental timeline. Participants viewed short humorous or neutral video clips (4000–16,000 ms) and were given 3000 ms following the clip to indicate with a button press whether they liked or did not like the video clip. Intertrial interval ranged from 500 to 13,000 ms.

The task consisted of a set of 32 short color video clips chosen from two categories: either funny (16 clips with average duration of 11 ± 2.06 sec) or neutral (16 clips with average duration of 10.18 ± 1.55 sec). Stimuli order were randomized. After watching each video clip, participants were asked if they thought the clip was funny by providing a “smiley face” and a “frowny face: and asked to respond either yes (happy) or no (frowny). Regardless of the initial categorization into funny or neutral, funny trials were counted as funny if the participant responded “Yes” (smiley face) and were counted as neutral if the participant responded “No” (frowny face). In order to have enough power to detect changes in activity, only participants with at least 10 trials in each category were included in the group statistics (this excluded 8 participants who, despite completing the task in full, chose either less than 10 trials as funny (N = 3) or less than 10 trials neutral (N = 5). This excluded 5 females and 3 males, average age of 6.79 (SD = 0.51), average IQ of 112.75 (SD = 13.13) and left a final sample of N = 27, female = 10, average IQ of 115.74 (SD = 14.51). Briefly, each trial started with a fixation cross that was presented for 500–13,000 ms (timing was based on optseq output) after which the video started and played for its full duration (4000–16,000 ms). After the video played, the participant had 3000 ms to respond. Post-response, the screen was replaced with a black background until the onset of the next trial (Fig 1).

### fNIRS acquisition

We measured cortical neural activation using a 42 channel NIRScout (NIRx Medical Technologies) fNIRS device with a sampling rate of 7.81Hz. A total of 32 (sources = 16, detectors = 16) fNIRS optodes were distributed over both hemispheres. We positioned the optodes over standard 10 to 20 system locations using individually sized caps (Brain Products) selected based on head circumference. The 10 to 20 locations were spatially adjusted across all cap sizes to maintain consistent coverage of our regions of interest despite changes in head size across participants [33]. Consistent 3-cm channel distance was achieved using plastic supports between each source/detector pair that constituted a recording channel.

**Fig 2:**
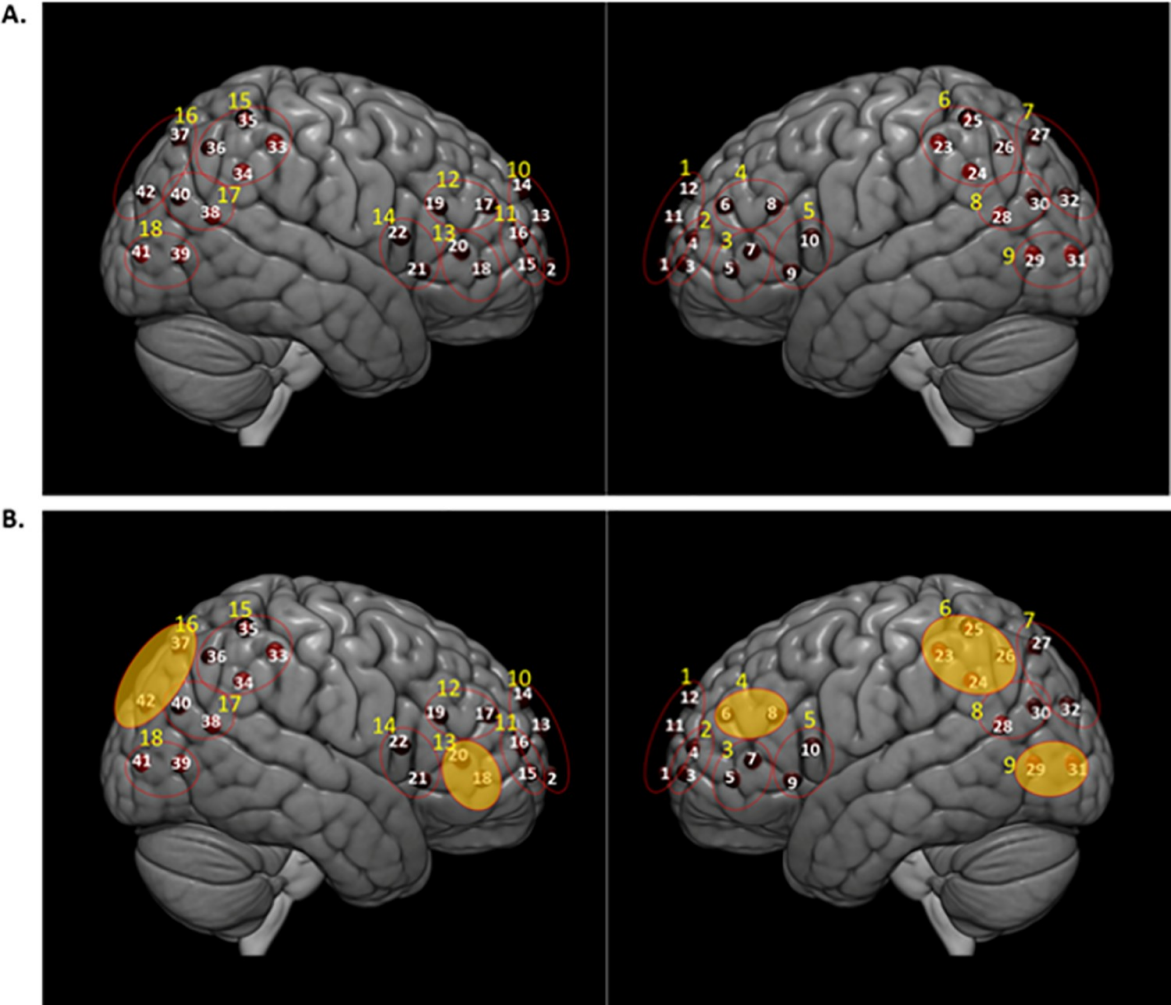
(A) fNIRS channel locations and functional localization into 18 ROIs based on proximity of channels and anatomy. White numbers represent channels and yellow numbers represent ROIs which include 1 = left frontopolar cortex; 2 = left middle frontal gyrus; 3 = left inferior frontal gyrus BA 47 and 45; 4 = left dorsolateral prefrontal cortex; 5 = left inferior frontal gyrus BA 44 and 45; 6 = left inferior parietal lobe; 7 = left superior parietal lobe; 8 = left temporoparietal junction; 9 = left temporo-occipito-parietal junction; 10 = right frontopolar cortex; 11 = right middle frontal gyrus; 12 = right dorsolateral prefrontal cortex; 13 = right inferior frontal gyrus BA 47 and 45; 14 = right inferior frontal gyrus BA 44 and 45; 15 = right inferior parietal lobe; 16 = right superior parietal lobe; 17 = right temporoparietal junction; 18 = right temporo-occipito-parietal junction. (B) fNIRS funny>neutral contrast significant results (p<0.05 FDR corrected). ROIs that exhibited both HbO and HbR significant changes for funny>neutral (p<0.05 FDR corrected) included left DLPFC (ROI 4), left IPL (ROI 6), left TOPJ (ROI 9), right IFG (ROI 13) and right SPL (ROI 16).

Our montage was designed to optimize coverage of brain structures in the frontal, temporal, and parietal lobes. Fig 2 shows the channel locations as well as the functional localization clusters (described later).

### fNIRS data analysis

All preprocessing and analysis of fNIRS data was conducted using the HomER2 package in Matlab. First, all optical density data were corrected for motion artifacts by the use of a wavelet motion correction procedure [34]. Next, the optical density data were bandpass filtered between 0.01 and 0.5 Hz prior to being converted to oxygenated hemoglobin (HbO) and deoxygenated hemoglobin (HbR) values using the modified Beers-Lambert law [35]. Individual fNIRS channels were rejected for the following reasons: 1) change in signal to noise ratio (SNR) measured by the Homer2 ‘enPruneChannels’ function (i.e., ±2 SD change in SNR); or 2) critically low signal quality based on NIRx calibration methods (i.e., >10% of scan demonstrating high (>2.5) or low (<0.03) signal voltage or >7.5 SNR). Data were excluded at the level of the region of interest.

### Statistical analysis

#### General linear model analysis of cortical activation

We assessed patterns of brain activation using a generalized linear model (GLM) approach, an approach that has been well established for analysis of event-related as well as blocked fNIRS designs [36, 37]. The GLM procedure assumed a canonical hemodynamic response function and Gaussian error structure. The onset and duration of each condition of interest (funny and neural) were submitted to the GLM procedure as predictor variables used to estimate standardized β coefficients for each condition and within each channel. The sign and magnitude of each β coefficient provides an indicator of the direction (positive/negative) and intensity of blood oxygen level-dependent change (i.e., brain activity) that occurs during each condition. In order to capture the brain activation unique to the task demands, and thus not expected to be present in signals corresponding to the control condition, we made contrasts between each β coefficient and its corresponding control. The outcome of these contrasts were then submitted to the functional localization procedure described below.

### Functional localization

We utilized a functional localization approach [37, 38] to account for variation in cortical activation in response to our tasks. This procedure allows for minor individual variation in the location of task-responsive brain regions across participants and reduces the risk of committing type II (i.e., false negative) errors due to averaging across nonresponsive channels. We grouped channels based on proximity and anatomical location (Fig 2) to create 18 clusters (9 in each hemisphere). Within each of the 18 functional localization clusters, we identified the single channel that responded greatest to each condition by selecting the channel with the greatest beta contrast (result of the single subject GLM analysis as explained above). The localized channel within each region of interest was then submitted for group-level statistical analysis.

### Group analysis

We used 1-sample t tests to determine whether there was significant brain activation (increased HbO and decreased HbR) in each localization cluster for our contrast of interest (Funny vs. Neutral, FDR corrected for multiple comparisons). HbO of clusters showing significant effects were entered into a secondary correlation analysis (not corrected for multiple comparisons) with behavioral measures, including age, personality measures and IQ. The change in oxygenated hemoglobin level is considered to be a good indicator of brain activity [[39, 40] but see also [40]]. In addition, gender differences were assessed using one-way ANOVA.

### Connectivity analysis

Wavelet Transform Coherence (WTC) was used to assess coherence between regions. WTC can identify locally phase locked behavior between two time-series by measuring cross-correlation between the time-series as a function of frequency and time [41–43]. WTC is well suited to investigate non-stationary changes in coupling between fNIRS time-series and has been used to identify both intra- and inter-brain dynamics across multiple tasks. For a more in-depth explanation of WTC, please see Grinsted, Moore, and Jevrejeva [44] and Chang and Glover [45]. The WTC package in Matlab [44] was used. For each ROI the representative channel based on the functional localization method described above was used, resulting in 18 representative channels representing the 18 ROIs. WTC was calculated for all these channel pairings. The optical density data entered into each WTC decomposition were unfiltered. In order to normalize the statistical distribution of the values within each matrix of coherence values, each value was subject to Fischer z-transformation [46, 47]. Next, for each condition of interest, the data were reduced to the condition-relevant frequency band and time. Based on our task structure, the frequency band of interest was between 10s and 25s, corresponding to frequency 0.1Hz and 0.04Hz respectively. The mean of each condition-relevant data subset was then calculated. Averaged WTC was calculated for all channel pairings within the left PFC (ROIs 1,2,3,4,5), right PFC (ROIs 10,11,12,13,14), left parietal (ROIs 6,7,8,9) and right parietal (ROIs 15,16,17,18) (e.g., between all channels within the left prefrontal region of interest). In addition, averaged WTC was calculated for channel parings within each hemisphere’s PFC and parietal regions (for left PFC-parietal between ROIs 1,2,3,4,5 and 6,7,8,9; for right PFC-parietal between ROIs 10,11,12,13,14 and 15,16,17,18). The averaged coherence values for these 6 regions were submitted for statistical analysis.

### IQ and personality assessments

In order to assess personality, a well-established assessment of personality traits was given to parents to fill out online. The Inventory of Child Individual Differences (ICID; [48]) was developed specifically to measure these traits in children. The ICID measures five higher and 15 lower order traits. The five higher order traits are analogous (but not identical) to the Big Five in adult populations, which are Neuroticism (the tendency to experience negative emotions, such as anger, anxiety, or depression), Extraversion (the tendency to be characterized by positive emotions, surgency, and to seek out stimulation and the company of others), Openness to Experience (the tendency to appreciate art, emotion, adventure, unusual ideas, imagination, curiosity, and variety of experience), Agreeableness (the tendency to be compassionate and cooperative rather than suspicious and antagonistic toward others), and Conscientiousness (the tendency to show self-discipline, act dutifully, and aim for achievement).

The WASI-II was used to measure general intelligence. The measure consists of 4 subtests, Vocabulary, Similarities, Block Design, and Matrix reasoning, which are used to estimate Full Scale IQ (FSIQ). The WASI-II has a mean of 100 and a SD of 15.

## Results

### Behavioral results

Average percent of “Funny” choices for funny videos was 80.78% (SD 11.8) and “Not Funny” choices for neutral videos was 85.87% (SD 11.95). There were no differences between females and males on funny choices or reaction times (p values from p = 0.55 to p = 0.87) and no correlation with IQ. Age was negatively correlated with reaction times (r = -0.435 p<0.05 for funny choices and r = -0.499 p<0.01 for neutral choices). There were no significant differences between females and males on any of the personality measures or IQ levels.

### fNIRS data

We first computed the main effect of funny>neutral. Regions that exhibited both HbO and HbR significant changes for funny>neutral (p<0.05 FDR corrected) included left dorsolateral prefrontal cortex (DLPFC; ROI 4), left inferior parietal lobe (IPL; ROI 6), left temporo-occipito-parietal junction (TOPJ; ROI 9), right inferior frontal gyrus (IFG; ROI 13) and right superior parietal lobe (SPL; ROI 16) (Fig 2, Table 1). There were no significant effects of sex.

**Table 1:** Beta contrast of activation for funny>neutral for regions surviving FDR correction of p<0.05 for both HbO and HbR.

	HbO			HbR		
Region	HbO Beta	95% CI	p	HbR Beta	95% CI	p
Left DLPFC	4.43	(2.02,6.84)	0.004	-5.54	(-8.71,-2.37)	0.001
Left IPL	10.13	(7.78,12.48)	0	-8.98	(-11.84,-6.11)	0
Left TOPJ	5.58	(2.65,8.52)	0.001	-6.92	(-10.26,-2.63)	0.002
Right IFG	4.17	(1.83,6.52)	0.001	-4.04	(-6.97,-1.10)	0.009
Right SPL	4.86	(2.22,7.50)	0.001	-3.79	(-6.55,-1.03)	0.009

**Fig 3:**
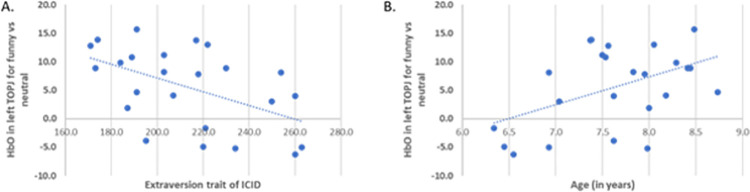
Correlation analysis with HbO and behavioral indexes. (A) HbO in left TOPJ negatively correlated with extraversion (B) HbO in left TOPJ positively correlated with age.

We next computed correlation effects between activation patterns in the funny vs neutral conditions in these ROIs and age and 5 personality traits from the ICID (Fig 3). None of the correlations survived FDR correction for multiple comparisons. However, as planned a priori, the uncorrected analyses were examined with the goal of hypothesis-generation for future studies. Activity in left TOPJ was positively correlated with age (r = 0.468 p<0.05, uncorrected) and negatively with extraversion trait of the ICID (r = -0.508 p<0.05, uncorrected).

### fNIRS coherence data

**Fig 4:**
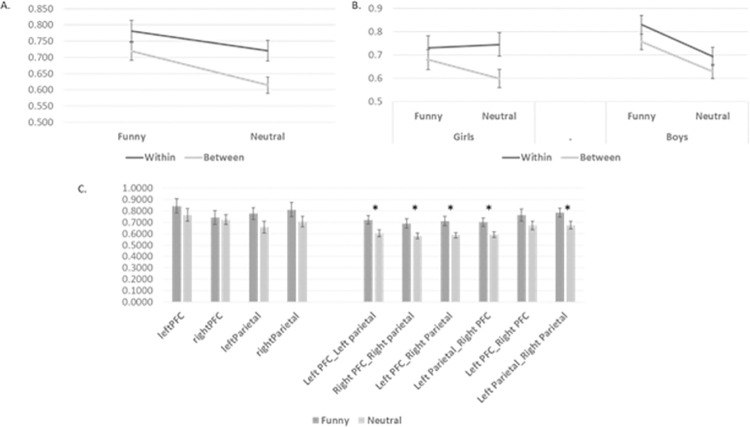
(A) Coherence analysis between and within regions (B) Coherence analysis between and within regions separated for girls and boys (C) Paired t-test analysis between funny and neutral conditions. * significant result at p<0.05 FDR corrected.

In addition to activation differences, we were also interested in testing connectivity between regions. Four larger regions depicting left/right PFC and left/right parietal regions were identified consisting of subregions 1,2,3,4,5 for left PFC, 10,11,12,13,14 for right PFC, 6,7,8,9 for left parietal and 15,16,17,18 for right parietal. In order to assess within- and between-region coherence, all coherence values were first codified based on their coupling–either within or between these larger regions. and averages were created for each coupling. A repeated measures ANOVA was conducted with condition (funny, neutral) and coupling (within, between) as repeated measures. This analysis revealed a significant effect for condition (p<0.001, partial η2 = 0.26, with funny exhibiting larger coherence values), and coupling (p<0.0001, partial η2 = 0.62, with within-ROI exhibiting larger coherence values). When entering sex (girls, boys) as a between group factor, there was a significant three-way interaction of sex BY condition BY coupling (F = 4.85, p<0.05, partial η^2^ = 0.16). While boys exhibited significant differences in condition (p<0.01, partial η^2^ = 0.53) and coupling (p<0.0001, partial η^2^ = 0.69), girls exhibited an interaction between these measures due to comparable coherence values for funny and neutral in the within-region analysis (see Fig 4). Thus, while both girls and boys exhibited elevated coherence values within ROIs compared to between ROIs, there was no difference between humorous and neutral content within ROIs for girls.

To further investigate the within regions effect seen, we conducted separate paired t-tests between funny and neutral condition for all 4 regions (left PFC, right PFC, left parietal and right parietal) for all within and between regions coupling. We found significant differences between funny and neutral in all couplings involving coherence between PFC and parietal regions, all surviving p<0.05 FDR correction: the left PFC-left parietal (t = 3.27, d = 0.87), right PFC-right parietal (t = 2.58, d = 0.68), left PFC-right parietal (t = 3.26, d = 0.86) and left parietal-right PFC (t = 2.81, d = 0.75) couplings. We further investigated this effect by repeating this analysis separately for boys and girls. For boys there were significant differences between conditions within left parietal region (t = 2.21, p<0.05, d = 0.54), right parietal regions (t = 2.24, p<0.05, d = 0.55) and between regions left PFC-left parietal (t = 2.52, p<0.05, d = 0.65), right PFC-right parietal (t = 2.39, p<0.05, d = 0.61), left PFC–right parietal (t = 2.66, p<0.05, d = 0.70), left parietal- right PFC (2.48, p<0.05, d = 0.64). There were no significant effects for girls indicating that the whole group effects seen might be largely driven by the boys (Fig 4).

## Discussion

The goal of the current study was to extend our knowledge about the neurodevelopmental profile of humor appreciation. Specifically, we sought to extend previous work looking at the neural correlates of humor development in younger children. We chose to focus on a relatively small age range, between 6 and 8 years old. Our results replicate much of the same regions observed in previous studies to be related to humor appreciation, including left TOPJ, IPL, DLPFC and right IFG and SPL. In addition, we extend previous results by showing age related changes in TOPJ activation and differences between female and male in connectivity patterns in the right fronto-parietal network.

In this study, we did not use a non-humorous positive stimulus that could be useful for dissociating reward-associated activation from activation related specifically to humor processing [26, 28]. While this is a limitation of the current study, we choose to focus on the cognitive component of humor, which is better represented in cortical activations (compared to sub-cortical areas, which cannot be directly interrogated using fNIRS). This choice allowed us to maintain a shorter and more manageable study design suited for younger children while still maintaining good power for planned statistical analyses. Future studies should test the specificity of these results to feelings of mirth and disassociate them from positive feelings in general.

### Activation patterns

One of the most consistent findings in humor research is observation of TOPJ activation in many types of humor processing in both adults and children [24, 26, 49]. The TOPJ is an anatomical hub at the intersection of occipital, temporal and parietal lobes. As such, its involvement in humor processing has been taken to signal the need for integration of information for incongruity detection and resolution [49, 50]. While here we report on left lateralized TOPJ activation for funny vs neutral clips, previous studies have found bilateral activation of TOPJ in processing humor stimuli among children [26]. Lateralized left TOPJ involvement in humor processing has been reported in adult studies of humor processing [23], suggesting a developmental lateralization with age. We found that activation of left TOPJ in response to funny stimuli was correlated with age (although only as a trend in an uncorrected analysis), so that activation was stronger in older children. It’s interesting to note, that our age range was relatively small (6.1–8.7 years). The fact that even with this limited age variability we still see activation increases with age is in line with research showing exponential maturation of brain regions and white matter tracts from 5 years into early adulthood [51, 52]. Specifically related to the TOPJ, white matter coherence in the inferior fronto-occipital fasciculus has been reported to increase exponentially from childhood to early adulthood [51]. The inferior fronto-occipital fasciculus is a white matter tract connecting frontal areas (including the inferior frontal gyrus and prefrontal regions) to posterior temporal, occipital, and superior parietal regions [53].

Age related changes in humor appreciation have been documented in several studies. These changes likely reflect developmental milestones such as the ability to comprehend cause and effect, language development and the ability to classify and categorize concepts [3, 54–57]. Some theories posit that children below age 6–7 while being able to detect incongruities, do not necessarily understand the resolution to that mismatch even when it is hidden in the joke [3, 58]. According to this view, preschoolers usually find an incongruous event/joke funny mainly because it makes no sense to them, and not due to the ability to resolve the incongruency created by the event/joke [3, 58]. Other researchers point to the fact that stimuli used, especially in this young age group, can account for these results and that when the content is presented more visually rather than verbally, children of 4- and 5-year-old appreciate the resolution information of an incongruous simplified event/joke [54]. The age-related results observed here (although uncorrected for multiple comparisons) and in other studies in the left TOPJ might be due to different developmental abilities related to the ability to detect and resolve incongruity, and varying ways that these children might have processed the humorous content. While some children were able to understand the incongruity and resolve it, others might have enjoyed the incongruity without requiring its resolution.

In the current study, we did not find any effects of sex on activation patterns resulting from the GLM analysis. Connectivity patterns did vary between the sexes as is discussed below. Lack of activation differences between girls and boys is in contrast to previous work that reported sex differences in processing of humorous content in bilateral temporo-occipital cortex [27]. One distinction between previous and the current study is that while Vrticka et al., (2013) compared funny and positive (but not funny) stimuli, in the current study we focused on funny vs neutral stimuli and did not present positive (not funny stimuli). By doing so, we might have missed these sex differences. The Vrticka et al., (2013) study also utilized fMRI while this study used fNIRS.

In addition to left TOPJ, we also observed increased activation in left IPL and DLPFC for our main contrast of interest. Left IPL activation has previously been reported for positive>neutral contrast [26] as well as for funny>neutral [27]. IPL activation has also been reported when contrasting incongruity resolution to incongruity identification [6]. This is in line with results of the current study as well as previous studies in children [26, 27]. Thus, it appears that both the left TOPJ and IPL may play important roles in the element of incongruity detections (TOPJ) and resolution (IPL) which are cornerstones of humor appreciation [59]. The left DLPFC is part of the executive control network, which is implicated in emotion regulation [60]. Emotion regulation, as part of humor appreciation, can be thought as reflecting automatic emotion regulation during humor processing [50]. Kohn et al. (2011) reported increased DLPFC activation coupled with hippocampus and superior frontal cortex activation during viewing of humorous cartoons. These activations were taken to indicate an important role of emotion regulation in humor appreciation.

In addition to this left lateralized network, right IFG and SPL were also significantly activated during humor appreciation. Both the right IFG and SPL are related to processing of remotely associated ideas. For example, right IFG activation has been seen in processing novel metaphors compared to conventional, which requires creating novel semantic connections between remotely associated words and searching for new meaning [61]. Right SPL has been seen to be activated in tasks requiring access to a large semantic space (compared to a constrained space), which includes demands related to hypothesis generation, semantic retrieval, semantic categorization, and cognitive monitoring [62]. Together, activations seen in the current study, relating to humor appreciation in both right IFG and SPL might indicate that humorous content of funny videos, compared to the neutral ones, required participants to search meaning in a large semantic network. Indeed, humor appreciation, through the incongruity-resolution model, involves access to ideas that are remotely associated in order to process the incongruity that leads to the feeling of mirth [59, 63, 64].

### Coherence analysis

In addition to activation patterns related to humor appreciation, we were interested to assess connectivity patterns in connection to humor. Overall, we found that within-region coherence, across our task, was greater than between-region coherence. That is, each channel within a region correlated highly with each of its neighbors within the same region in response to our task (both for the funny and neutral conditions). A similar pattern of increased coherence within spatially proximate fNIRS channels has also been reported in resting-state connectivity analysis [47, 65] and task related coherence analysis [37]. For example, Medvedev [47] identified greater connectivity among channels within compared to between anatomical region of interests (inferior frontal gyrus and middle frontal gyrus). This pattern of increased coherence within neighboring compared to remote fNIRS channels might be related to cerebral blood flow autoregulatory processes that vary significantly between large regions of the brain but are similar within neighboring brain regions [66].

We also observed increased coherence in response to humorous content compared to neutral content, primarily between regions. Previous studies have identified interhemispheric and frontoparietal increases in connectivity from resting state to task performance which increased as the mental load of the task increased [67–69]. This might indicate that functional connectivity may provide a unique indicator of mental effort. In the context of the current results, this suggests that humor processing imposes greater mental load than neutral video viewing and that viewing humorous videos requires more information processing than neutral videos.

It is interesting to note that this pattern of results was more pronounced for boys than for girls in our population. Girls did not show a difference between conditions in connectivity when looking between regions, while boys exhibited stronger coherence between frontal and parietal regions as well as within regions for the humor condition (Fig 4). One possibility is that these results are due to the humorous content of our stimuli and how boys and girls in our cohort experienced them. While there is evidence that males and females find different content humorous [70, 71], our behavioral results suggest that the boys and girls in this study did not differ in their subjective ratings of the videos. Another possibility is that these results reflect different neuro-developmental trajectories in boys and girls not related to the humorous content. Wu, Taki [72] examined topological organization of functional brain networks derived from resting state fMRI in healthy children. They found effects of age, sex, and their interaction, indicating that girls and boys have distinct developmental patterns of functional brain networks. Boersma, Smit [73] examined whole brain connectivity and network topology using electroencephalography (EEG) in children between the ages 5–7. They found significant differences between girls and boys suggesting that girls have stronger whole-brain connectivity and more ordered network topology than boys at this young age. As our cohort of participants fall in a relatively small age range (6–8 years old), an interesting future direction would be to conduct a longitudinal study to test the possibility presented above, that humor appreciation is related to different neuro-developmental trajectories in girls and boys.

### Limitation

The current study has several limitations. While fNIRS is a feasible alternative to fMRI and actually offers several advantages, for example, by providing the opportunity to conduct studies in an ecologically valid study design setting, it is also limited to measuring hemodynamic changes on the cortical surface. Thus, while we were able to capture TOPJ and IFG activations, we were not able to capture deep brain structures related to reward. We chose fNIRS as a more comfortable imaging method than fMRI and with better spatial resolution and less susceptibility to head movement compared to EEG [74] due to the young age range of our participants, but encourage future studies to include this age group in functional imaging studies able to capture activation in deep brain regions. Other limitations are related to the stimuli used. In this study, we did not use a non-humorous positive stimulus, that could be useful for disassociating reward-associated activation from activation related specifically to humor processing [26]. While this is a limitation of the current study, we choose to focus on the cognitive component of humor which is better represented in cortical activations (compared to sub-cortical areas, which cannot be directly interrogated using fNIRS), thus allowing us to maintain a shorter and more manageable study design suited for younger children while still maintaining good power for statistical analysis of brain activation. Future studies should test the specificity of these results to feelings of mirth and disassociate them from positive feelings in general. Another limitation is related to the potential variance in our stimulus set due to the temporal nature of our video clips. While cartoon jokes could allow for better methodological control, using video clips was more compatible with our developmentally young cohort, requiring less cognitive effort to process. It is also possible that the type of humor featured may have influenced activation [24]. Future studies might use a more temporally detailed video and behavior capturing device able to discern the exact moment of peak humor. In addition, motion related to laughter might have a confounding effect causing increased motion artifacts. In order to account for this, we both corrected for motion artifacts using wavelet motion correction procedure as well as visually surveyed all channels across all participants and flagged ones that were deemed as noisy. However, future studies could include an external measure of laughter motion to specifically account for this potential factor. In the current study we focused on analyzing humor perception and appreciations as a whole event, and did not look into the time varying processes involved in humor processing, such as suggested by Suls [4] incongruity–resolution theory. According to this theory, humor appreciation has at least two stages where first incongruity needs to be recognized and then resolved. This is best exemplified by jokes that have a setup and a punch line, leading to the subsequent resolution and eventual feeling of mirth [2]. Future research that investigates time varying temporal processes may be able to provide a more fine-grained understanding of humor processing, especially as it pertains to development.

## Conclusions

The primary aim of the current study was to better understand neurodevelopmental trajectories of humor comprehension and appreciation in young children. While many studies have been conducted among adults, there is limited information in children. Using fNIRS we were able to both replicate previous findings of TOPJ and IFG involvement in humor appreciation in children obtained with fMRI and emphasize the association with age and gender differences in development. While the current work expands our understanding on the neurodevelopment of humor as a crucial part of heathy development, we encourage future work to further characterize how and why children vary in their ability to comprehend and appreciate humorous content.
